# On the Treatment of Cancerous Diseases

**Published:** 1862-11

**Authors:** E. Marguerat

**Affiliations:** Chicago


ARTICLE XXXVIII.

### ON THE TREATMENT OF CANCEROUS DISEASES.

By E. MARGUERAT, M.D., of Chicago.

Read before the Chicago Medical Society, 1862.

I am aware, gentlemen, that this is rather an unpromising subject for an essay now a days. The incurability of cancers has been so often proclaimed, *ex cathedra*, that late writers have become very dogmatic on the subject. Still, for my part, I like the hopeful spirit of old Dr. MOTT, who, so often used to say in his leetures, that he had no doubt but some day a specific should be found for the cure of consumption and cancers. It has been frequently reproached to some of our late clinical writers, especially in France, that they display all their talent and energy for the improvement of pathology and diagnosis, but scarcely give a passing attention to the treatment. This is, perhaps, too much the case to-day with the profession at large, on the subject of cancers. As a general rule, when a cancerous patient applies to a medical man, the knife is the only hope offered to him, and if he is too timid, or too incredulous to submit to an operation, his only resourse is in the advertising charlatan, and it is my candid opinion that he is not always successful there. The *ipse dixit*, ought to have less power in the practice of medicine than anywhere else; prejudice has often barred the way to progress. Because we have no medicine that has proved generally successful in the treatment of cancers, authors have denied in toto that any case of cancer had ever been cured by medicine.

It appears, unfortunately, that we have no rule by which we can diagnose with certainty a cancerous growth in its first stage at least. Even the microscope fails to give us a certain criticism. The so called cancer-cell on which so much importance has been placed, has lost a part of its claims. VIRCHOW, in his late work on cellular-pathology, comes to the conclusion that cancerous formations have no tissue-element that belong to them exclusive. Hence the great difficulty that has always been experienced in defending their claims, by those who believed to have cured cases of cancer. It has always been an easy matter to accuse them of error in diagnosis. The case was cured, ergo, it was not a case of cancer. There is, however, one class of so called semi-malignant growths, for which a little more charity has been shown. A great deal has been written of late years on epithelial cancers, epitheliomas, or canceroids; a seperate class has been made of them, not however on a very sure foundation. Many prove to be as truly malignant in their nature as carcinoma; and to quote VIRCHOW again: "It is impossible to find a well defined limit between the different formations of this group; they might all be included under the collective name of cancers, but for the clinical experience which shows that cancers generalize themselves usually, while cancer-oides do so seldom. The malignancy of new formations, depends much less on their tissue elements, than on the following two considerations: their more or less degree of organization, and their state of fluidity.

However this may be, it must be admitted that cancers are as much a constitutional as a local affection; hence, the constitutional treatment ought to have, at least, as much importance as the operative treatment. PAGET, in one of his papers, gives the statistics of a large number of cases, from which he concludes that in cases of scirrhus of the breast, patients die on an average thirteen months sooner, on whom an operation has been performed. If such should be the case, it is well worth the while to see what has been done; and what can be done, by a medical treatment. Dr. COOKE, in his essay, read before the London Medical Society in 1858, says, that the constitutional treatment of cancers is the only one in which he has any confidence. He places his chief reliance on a proper regulation of the diet, and habits of the patient, together with a tonic medication.

He claims to have by a persevering attention to this system, completely eradicated some well marked cases of cancerous diathesis.

Dr. TEESON, surgeon of the Middlesex Hospital, reports a case of mammary scirrhus entirely cured by the use of chlorid of zinc, both internally and externally; and another case much benefited by the same treatment.

Dr. MONTGOMERY, in his essay on cancer of the womb, terminates by saying that he knows such cases to be curable without operation, if taken early. Antiphlogistic treatment at the beginning, followed by iodine, iron, arsenic, with local application of nitrate of silver, when necessary, form his treatment.

Dr. WALSHE relates a case of incipient tumor of the breast, that completely and permanently disappeared under compression, and the internal administration of iodide of arsenic.

Dr. FANCHOU, in 1849, presented to the Medical Society of Paris, two cases that were admitted by them as cancerous. After a three month's treatment, the first case, a cancer of the chin was returned cured; the second, a mammary scirrhus in its second stage, was very materially benefited. His remedies were iodine and arsenic, internally and externally, with the necessary attention to diet, etc.

LANDLOFI, a surgeon of the metropolitan army, submitted in 1855, to a commission of physicians both in France and Germany, the result of his treatment of several cases, acknowledged by all, as cases of a malignant nature. Several of them were in succession returned, either cured, or materially improved, by the persistent use of chlorid of bromine. Among the cases are two cancers of the breast.

It would be tedious to repeat at length all the authorities on the subject. STORK, HAVELAND, STANLEY, SPENCER, WELLS, SIMON, DEVAY, MANCE, all men of reputation and integrity, report cases successfully treated by them. One case related by Dr. A. T. THOMPSON, has a special interest; it is that of a woman, 46 years of age, with a double mammary tumor, offering all the characters of scirrhus. After a persevering use of iodide of arsenic and conium, for more than eleven months, the tumor had entirely disappeared, and the woman was in good health.

Many other authorities might be quoted, but these are sufficient for my purpose. They satisfy me, that a good deal can be done by medication in the cancerous diathesis; and I cannot subscribe to the sweeping assertion of ERICHSEN and others, "that all constitutional curative treatment of cancers is altogether useless. To be sure, in the present state of our knowledge, we have no remedies possessed of a specific virtue in the treatment of cancers; but we have in the bichloride of mercury, iodine, chlorine, bromine, arsenic, and their combinations, a valuable store that has power and will still prove useful. And, I think, if we had had more men of the perseverence of Dr. THOMPSON, we should be able to produce more cases of cures.

It has been my privilege to spend some time with Dr. MAYER, formerly a surgeon in the Hospital of Lausanne. During his short stay in a country district in the State of N. Y., he was called upon to treat several cases of cancerous diseases, and had at that time a large reputation for skill and success. Although an advocate of the operation in all cases that permitted it, he relied in many cases on a combination of external applications and constitutional treatment. He employed in turns iodine, arsenic, the bichloride, &c., in varying combinations with tonics; caustic ointments of a slow action, nitrate of silver, potassa, &c. I have no notes of any of his cases, but I can testify to many cases of malignant ulcers entirely cured, and several cases of cancer of the breast improved by him.

My own experience has furnished me with a few cases that have been of great interest to me. I shall only briefly report a few. The first was a case of cancer of the lower lip, that I followed under Dr. Mayar's treatment. The cancer had commenced as a wart, on the side of the lower lip, and had gradually gone to ulceration. The patient was a man about 50 years of age. He had been treated by several physicians; had apparently taken a good deal of arsenic, without benefit, when he applied to Dr. Mayar. As he was not willing to submit to an operation, he was put on the use of iodine internally, with occasional cauterization of the ulcer, anodyne plasters, and bichloride of mercury. Dr. Mayar, at that time, returned to Europe, and left the patient in a rapidly improving condition. At the end of three months' treatment, the ulcer was cicatrized and the patient well.

The second case is that of Mr. C. Jackson, Lansing, N.Y. Several years before I saw him, he had been troubled with a chronic form of ulceration of the cheeks, nose, and eyelids. He had been under the care of several cancer curers. When he applied to me, two years ago, he had half a dozen ulcers, of the size of a ten cent piece, on his cheek; a hard indolent swelling of the upper lip, and the right ala nasi was perforated by an ulcer, through which I could pass the end of my finger. The skin around the ulcers was red and hard. He was a man 70 years old, of temperate habits; had never had any syphilitic disease. I first subdued the inflammation by cooling emolient applications and rest, and after a while put him on the use of Donovan's solution. I then applied chloride of zinc to the ulcer in the nose, and nitrate of silver repeatedly on the ulcers of the cheeks. However, I obtained no benefit from external application for the first six weeks or two months; the ulcers kept growing, and that in the nose had nearly doubled in size. I kept increasing the dose of Donovan's solution until he complained of weakness and swelling in his limbs. I then gradually diminished the dose, and commenced again the application of chloride of zinc. I was very gratified to find after one application the ulcer in the nose take a rapid turn, assume a healthy gradulating appearance, and finally, entirely close, after three or four weeks; leaving only a puckered cicatrix. I kept him on infusion of sarsaparilla, *ad libitum.* The skin all over the face assumed a smooth and healthy aspect; the swelling of the lips disappeared; no return of the disease had occurred when I left a year ago.

I do not claim this case to be positively of a cancerous nature; but call it lupus, or canceroides, or anything else. It certainly was a case of a very obstinate character; and on it I have two remarks to make here: 1st, that no benefit in the treatment followed the use of caustics, until the constitutional treatment had been carried very far; and, 2d, that during the course of the treatment I happened to prescribe a few pills containing half a grain of calomel each. When he returned to me after having used them, the ulceration had spread at a rate, at least, twice as rapid as it had at any other time before. I could discover no other cause for it than the calomel; this again proves the importance of the constitutional treatment.

Five years ago, in Ithaca, N. Y., I saw in connection with Dr. CORYELL, a distinguished practitioner in the place, two cases of mammary cancers in their second stage, and very similar in their symptoms and history. Both presented a large ulcerated surface; the glands in the axilla and neck were in both enlarged and indurated. The cancerous cachexia was not yet very apparent. They were evidently not cases for operation. We undertook to do what we could, in the way of palliative treatment. Bichloride of mercury and arsenic were prescribed, in combination with anodynes; careful partial cauterization of the ulcers, followed by soothing applications, and tincture of iodine on the enlarged glands, were alternately used. After a few weeks' treatment, one of the patients tired of the process, yielded to the persuasions of some friends, and went to undergo an operation in New York City. After a few weeks' absence, she came back apparently perfectly cured, and greatly elated. Two weeks afterwards she died from some pulmonary affection, I believe. In the other patient, a perseverance in the treatment brought gradually a diminution in the size of the gland; the ulceration remaind nearly stationary; her general health kept unimpaired, and she was still alive when last I heard of her, a year afterwards.

I have no doubt, gentlemen, that most of you might draw from your experience cases similar to these, and, perhaps, of a still greater interest. Whatever conclusion you may have come to, I think I am justified in saying that all curative constitutional treatment is not altogether useless; and, whenever I meet with a case favorable for it, I shall try what a persevering constitutional treatment of cancer can do.

According to Dr. Fanchou's statistics, the number of cancers is on the increase. And, still in 1840, the number of deaths by cancer, in the city of Paris alone was nearly 800. It then comes within the province of every physician, to give a serious attention to the treatment of an affection that must often come under his care in the course of a long practice. It is only the general experience of the profession that can satisfactorily solve the question of the curability of cancers. It is an interesting fact connected with the history of cancers, that the first case on record, 900 years B.C., is reported by HERODOTUS, to have been cured without operation.

